# *Rhizoglomus intraradices* Improves Plant Growth, Root Morphology and Phytohormone Balance of *Robinia pseudoacacia* in Arsenic-Contaminated Soils

**DOI:** 10.3389/fmicb.2020.01428

**Published:** 2020-07-10

**Authors:** QiaoMing Zhang, Minggui Gong, Kaiyang Liu, Yanlan Chen, Jiangfeng Yuan, Qingshan Chang

**Affiliations:** ^1^College of Forestry, Henan University of Science and Technology, Luoyang, China; ^2^College of Food and Bioengineering, Henan University of Science and Technology, Luoyang, China

**Keywords:** arbuscular mycorrhizal fungi, *Rhizoglomus intraradices*, *Robinia pseudoacacia*, endogenous phytohormone, root morphology, arsenic stress

## Abstract

Arbuscular mycorrhizal fungi (AMF) are known to improve the resistance of host plants against various heavy metal stresses. However, the arsenic (As) resistance mechanism of AMF-inoculated woody legumes remains unclear. In this study, black locust (*Robinia pseudoacacia* L.) seedlings were cultivated in potted soils inoculated with or without AMF *Rhizoglomus intraradices* under three different levels of As stress (0, 100, and 200 mg As kg^–1^ soil) over 4 months. The objective of this paper was to investigate the effects of AMF on plant growth, root morphology, and the content and ratio of endogenous phytohormones and soil glomalin under As stress condition. As stress toxicity suppressed the AM spore germination and colonization, plant growth, and the content of soil glomalin and changed the morphological characteristics of the roots and the balance of endogenous hormone levels in plants. However, *R. intraradices* inoculation improved the shoot and root dry weights, total root length, root surface area, root volume, and the number of root forks and tips across all As treatments. *R. intraradices* inoculation obviously decreased the percentage of root length in the 0- to 0.2-mm diameter class and increased those in the 0.5- to 1.0-mm and >1.0-mm diameter classes; the percentages in the 0.2- to 0.5-mm diameter class were less affected by *R. intraradice*s inoculation. The concentrations of the easily extractable glomalin-related (EE-GRSP) and total glomalin-related soil protein (T-GRSP) were higher in the of *R. intraradices*-inoculated seedlings than those in the non-inoculated seedlings. Furthermore, *R. intraradices* inoculation increased the concentrations of indole-3-acetic acid (IAA) and abscisic acid (ABA), but decreased the concentrations of gibberellic acid (GA) and zeatin riboside (ZR). The phytohormone ratios of IAA/ABA, GA/ABA, ZR/ABA, and (IAA + GA + ZR)/IAA in the *R. intraradices*-inoculated seedlings were lower than those in the non-inoculated seedlings. These results indicated that *R. intraradices* alleviated As toxicity in *R. pseudoacacia* seedlings by improving their plant growth, altering root morphology, regulating the concentrations and ratios of phytohormones, and increasing the concentration of soil glomalin. The results suggested that AMF-inoculated *R. pseudoacacia* seedlings would be a critical factor in successful vegetation restoration and soil development in As-contaminated soils.

## Introduction

Arsenic (As) is a poisonous metalloid and exists naturally in all types of the crust rocks, especially orpiment, realgar, and other ores (Li et al., 2018). For nearly a century, As accumulation in ecosystems has been getting worse due to excessive anthropogenic activities, such as the mining of As ores, smelting of metal, burning of fossil fuel, irrigating croplands with As-contaminated groundwater, and using As-based agrochemical and phosphate fertilizers (Chen et al., 2007; Salim et al., 2009; Srivastava et al., 2009). As-based compounds are often reported to be widely released in groundwater and soils in Asia, Europe, and North and South America (Andrade et al., 2015; Kalita et al., 2018). They can enter the food chain and cause many pathologies, including carcinogenesis (Li et al., 2016; Kalita et al., 2018). Exposure to excess As concentrations in soils generally disturbs a series of physiological, biochemical, and morphological processes in plants (Spagnoletti et al., 2016; Sharma et al., 2017). The physiological reactions to As toxicity in plants include inhibited seed germination, reduced growth and yield, decreased chlorophyll content and photosynthesis rate, senescence, and even death, which ultimately lead to vegetation degradation in high As-contaminated areas (Srivastava et al., 2009; Spagnoletti and Lavado, 2015; Spagnoletti et al., 2017). Hence, some remedial measures need to be taken to prevent even further environmental degradation.

Plants evolve a series of strategies to adapt or cope with the negative impact of heavy metal (HM) toxicity (Bilal et al., 2019). Root cap cells first perceive stress signals of HM toxicity and stimulate the rapid synthesis of endogenous phytohormones, such as abscisic acid (ABA), gibberellic acid (GA), indole-3-acetic acid (IAA), and zeatin riboside (ZR) (Ludwig-Müller, 2000). These hormones regulate many physiological processes of plants for adapting in unfavorable environments by effecting on plant nutrient absorption, root morphology, and the expression of metal transporters, in which the root morphology reflects the adaptation, health, and nutrition status of plants (Liu and Wei, 2019). Furthermore, plant growth and development are highly related to the synthetic ability, content level, and the ratio of phytohormones (Ludwig-Müller, 2000).

Arbuscular mycorrhizal fungi (AMF) are widespread root-symbiotic soil microorganisms in the phylum Glomeromycota (Schüßler and Walker, 2010). In the course of 400 million years of evolution, these endophytes gradually established mutualistic associations with 80% of terrestrial higher plants (Smith and Read, 2008). Throughout the AMF–plant symbiotic process, from arbuscular mycorrhiza (AM) spore germination to structure development within the roots, the AM extraradical and intraradical hyphae act as living bridges between soils and plants, and they provide the essential carbohydrates and water for supporting plant life cycle (Smith and Read, 2008). AMF also influence the root morphogenesis by regulating the activities of the root meristem, which is related to the strategies of nutrient absorption for host plants (Trotta et al., 2006). AMF alleviating HM toxicity in plants has been widely accepted and recognized since Gildon and Tinker (1981) first reported that AMF *Glomus mosseae* can help *Trifolium repens* seedlings enhance their stress tolerance in Zn- and Cd-contaminated soils. Under the HM stress condition, AMF also regulate the balance of the phytohormones by intervening in the communication of signals during the process of AMF–plant symbiotic association (Smith and Read, 2008; Li et al., 2018). Phytohormones, as a kind of endogenous plant signal, are the most sensitive responders in these processes, and they stimulate plants to show appropriate responses to adapting to HM stress (Ludwig-Müller, 2000; Délano-Frier and Tejeda-sartorius, 2008). Therefore, AMF contribute to building a productive, healthy, and sustainable ecosystem, with vegetation recovery in HM-contaminated areas.

AM colonization is found in some plant roots in As-polluted soils, and some evidence have shown that higher plants adapting to As stress are often associated with AMF (Leung et al., 2006; Chen et al., 2007; Li et al., 2018). Sharma et al. (2017) have shown that *Rhizoglomus intraradices* and *Glomus etunicatum* participate in the antioxidant defense system and thiol metabolism of wheat against As toxicity. Leung et al. (2006) also have revealed that AMF stimulate the phenolic defense system in host plants by producing thiol-like glutathione, and this plant metabolite detoxified As to its non-toxic form in As-contaminated soils under acidic conditions. AM symbiosis improves the As resistance of host plants. Two related universal mechanisms are “growth dilution effect” and “mycorrhizal immobilization,” which indicate that AMF increase plant nutrient absorption, transform inorganic As into less toxic organic forms, translocate As from host plants to the AM hyphae, and dilute As concentration by increasing plant biomass (Li et al., 2016, 2018). But the effects of AMF on the root morphology of woody legumes, endogenous phytohormones, and soil glomalin under As toxicity stress are still unknown.

The phytoremediation and land reclamation processes in As-contaminated areas initially make use of *Pteris vittata* as As hyperaccumulators, but this pteridophyte has a low growth rate and poor environmental adaptability, which limit its widespread use (Leung et al., 2006; Kalita et al., 2018). The use of As-resistant woody plants for revegetation prevents off-site migration of As-contaminated soils *via* wind erosion, and it reduces the As concentrations in soils by extracting As ions in plants. Black locust (*Robinia pseudoacacia* L.), as a fast-growing woody legume, shows good adaptability to various environmental stresses including barren, salt, cold, drought, and HM (Tian et al., 2003). Hence, it is probably deemed as an ideal candidate of dominant pioneer trees for vegetation recovery in As-contaminated areas (Rajkumar et al., 2012). Moreover, *R. pseudoacacia* can establish an intimate symbiotic relationship with AMF in HM-contaminated soils (Huang et al., 2019). Recent studies have shown that AMF *Funneliformis mosseae* alleviates the effects of lead (Pb) toxicity by improving the plant biomass, root activity, antioxidant enzymes, and photosynthesis in *R. pseudoacacia* seedlings grown in Pb-contaminated soils (Yang et al., 2015; Huang et al., 2019). However, only a few literatures have reported on AMF strengthening the tolerance of woody legumes against As toxicity, particularly the related underlying effects on root morphology, endogenous phytohormones, and soil glomalin. In this study, a pot experiment of *R. pseudoacacia* locust seedlings was performed under different As treatments, with or without *Rhizophagus intraradices* inoculation. The present study aimed to (1) reveal the dynamic changes of AM spore germination, fungal structure development, and the AM colonization rate with the culture (or colonization) process and (2) elucidate the underlying effects of AMF on plant growth, root morphology, endogenous phytohormones, and soil glomalin. The present study will contribute to our understanding of the mechanism of AMF enhancing the tolerance of *R. pseudoacacia* seedlings against As toxicity.

## Materials and Methods

### Soil Materials

The soils used in this experiment were collected from the top layer of the botanical garden in Henan University of Science and Technology campus in Luoyang, Henan Province, China. The main physicochemical properties of the soils were as follows: organic matter, 17.38 g kg^–1^; available nitrogen, 37.84 mg kg^–1^; Olsen phosphorus, 8.17 mg kg^–1^; available potassium, 103.47 mg kg^–1^; and pH, 7.8 (1:5 soil/water ratio). The extractable metal concentrations (in milligrams per kilogram) in the soils were as follows: As, 3.73; Fe, 4.82; Mn, 1.87; Cu, 0.21; and Zn, 1.02. The soil texture for this study included soil and sand (1:1, *v*/*v*) and was mixed thoroughly, passed through a 2-mm sieve, and then autoclaved at 121°C and 0.11 MPa for 2 h before use.

In order to prepare the As-contaminated soils, the soil texture was sprayed with Na_3_AsO_4_×12H_2_O solutions to obtain a gradient level of As concentrations: 0 (control), 100, and 200 mg As kg^–1^ soil. The As concentrations were based on both the results of a preliminary experiment and the Chinese environmental quality standard of As contamination in soils.

### AMF Material

AM strain *Rhizophagus intraradices* (N.C. Schenck & G.S. Sm.) C. Walker & A. Schüßler (BGC BJ09) was provided by the Institute of Plant Nutrition and Resources, Beijing Academy of Agriculture and Forestry Sciences, Beijing, China. *R. intraradices* spores were propagated in a pot culture with *Zea mays* L. using sterile sand under greenhouse conditions. Four months later, the AM colonization rate was assessed (65.8%). The *R. intraradices* inocula consisted of mycorrhizal root fragments, spores (23 spores per gram), extraradical mycelium, and sand.

### AM Spore Germination, Pre-symbiotic Fungal Growth, and Colonization Rate

The AM spores were extracted from the above AM strain according to wet sieving (Gerdemann and Nicolson, 1963), surface-sterilized in 1% NaOCl for 15 min, and rinsed in sterile distilled water (Zhang et al., 2017). AM life stages were investigated by using a modified “sandwich system.” The sandwiched spore membranes were composed of two pieces of cellulose ester membranes (Millipore TM, 50 mm diameter, 0.45 μm pore diameter). Fifty surface-sterilized spores were placed on one membrane, which was covered by another empty membrane. After the seedlings were grown for 15 days in pots, the sandwiched membranes were folded and placed into the soils around the roots. In this way, the AM spores had no direct contact with the *R. pseudoacacia* roots, but their growth was affected by the root exudates and other signal substances.

To determine the rate of AM spore germination and the number of hyphal branches, the sandwiched membranes were extracted from the pots for each seedling line at 10, 15, 20, 25, and 30 days post-inoculation (dpi). The folded sandwich membranes were gently pulled out and washed out the soil mixture with tap water. Then, the membranes were spread out on a culture dish, stained with 0.05% Trypan blue for 5 min, and destained with distilled water. AM spore germination and hyphal branches were observed by using a stereoscopic microscope (Olympus SZ2).

*R. pseudoacacia* roots were harvested at 30, 45, 60, 75, and 90 dpi. The roots were cut on 1-cm root fragments, washed with deionized water, and stained with 0.05% (*w*/*v*) Trypan blue in lactophenol according to the method of Phillips and Hayman (1970). The AM colonization rate was determined by using the grid-line intersect method (Giovannetti and Mosse, 1980). The AM colonization rate and arbuscule abundance were determined with an optical microscope (Olympus BX51) and calculated using the method of Trouvelot et al. (1986).

### Plant Growth and Root Morphology Analysis

Black locust (*R. pseudoacacia* L.) seeds were obtained from a mother tree growing at the campus of Henan University of Science and Technology (Luoyang, Henan, China). Plump and uniform seeds were selected, sterilized in 75% ethanol for 20 min, rinsed three times with deionized water, and finally dipped in sterile water for 24 h. The seeds were germinated in autoclaved wet sand at 28°C. The healthy seedlings were selected and transplanted into conical frustum plastic containers containing 1 kg of soil mixture, with one plant each pot.

For seedling transplantation, 30 g AMF inoculum was placed in each pot of mycorrhizal treatment 2 cm below, while the non-mycorrhizal pot was added 30 g of autoclaved inoculum. The seedlings were cultivated in a greenhouse with temperature of 20–35°C and relative humidity of 50–75% from April to July 2018. Each pot was supplemented with 50 ml Hoagland’s solution weekly (Hoagland and Arnon, 1950) and the soil moisture of 50% field capacity was maintained with deionized water by regular weighing.

*R. pseudoacacia* seedlings were harvested separately at 50, 60, 70, 80, and 90 dpi, with three replicates for each harvest. The adhering particles on the *R. pseudoacacia* roots were carefully washed with deionized water. Shoots and roots were cut separately for determining fresh weights, and then the shoots and roots were weighed after oven drying at 75°C for 36 h for dry weight.

Root morphology analysis was carried out on the three replicate *R. pseudoacacia* seedlings, and roots were washed with tap water and soaked in 50% ethanol. The roots were scanned at 300 dpi by an Epson Expression 1680 Pro scanner (Epson, Nagano, Japan). The following morphometric parameters were analyzed with the scanner supporting WinRhizo (version 5.0B) root analysis system software (Regent Instrument Inc., Canada): total root length, root surface area, root volume, average diameter, tip number, and number of forks.

### Phytohormone Determination

The ultraperformance liquid chromatography–tandem mass spectrometry (UPLC/MS–MS) method was used for measuring the phytohormone contents of IAA, GA, ZR, and ABA. The fresh roots (1 g) were washed out the soil mixture with tap water and then were ground in liquid nitrogen using a mortar. These phytohormone samples were prepared and purified according to the method of Lin et al. (2015) on an LC system (ACQUITY UPLC, Waters, Milford, MA) coupled with a Waters Quattro Premier XE mass spectrometer (Micromass, Manchester, United Kingdom). The parameters were analyzed by the method of Fu et al. (2012).

### Glomalin Determination

Glomalin in soils is quantified as glomalin-related soil protein (GRSP). Easily extractable glomalin-related soil protein (EE-GRSP) and total glomalin-related soil protein (T-GRSP) were analyzed according to the method of Wright and Upadhyaya (1996). EE-GRSP was obtained from 1 g dry soil in 8 ml citrate solution (20 mM, pH 7.0) for 0.5 h at 121°C, and T-GRSP was extracted from 1 g dry soil in 8 ml citrate solution (50 mM, pH 8.0) for 1 h at 121°C. Sample extraction was repeated four times using the same extraction process, and the supernatants were pooled together and centrifuged at 10,000 × *g* for 5 min. Protein content in the supernatants was analyzed by the Bradford dye-binding assay at 562 nm using bovine serum albumin as standard (Wright and Upadhyaya, 1996).

### Experimental Design and Statistical Analysis

This experiment consisted of six treatments and was set up in a completely randomized block arrangement with a 3 × 2 factorial design: (1) three As level in soils, i.e., 0, 100, and 200 mg kg^–1^ dry soil, and (2) AMF treatments, i.e., *R. intraradices* and a non-AM-inoculated control. Hence, each of the six treatments had three replicates, making a total of 18 pots (one seedling per pot). Significant differences among these treatments were evaluated by Tukey’s multiple range test. Standard deviations (SD) were also calculated, and the values are presented as the mean ± SD. Statistical analyses were carried out using the SPSS Statistics (IBM SPSS, Somers, United States).

## Results

### AM Spore Germination Rate and Number of Hyphal Branches

**FIGURE 1:**
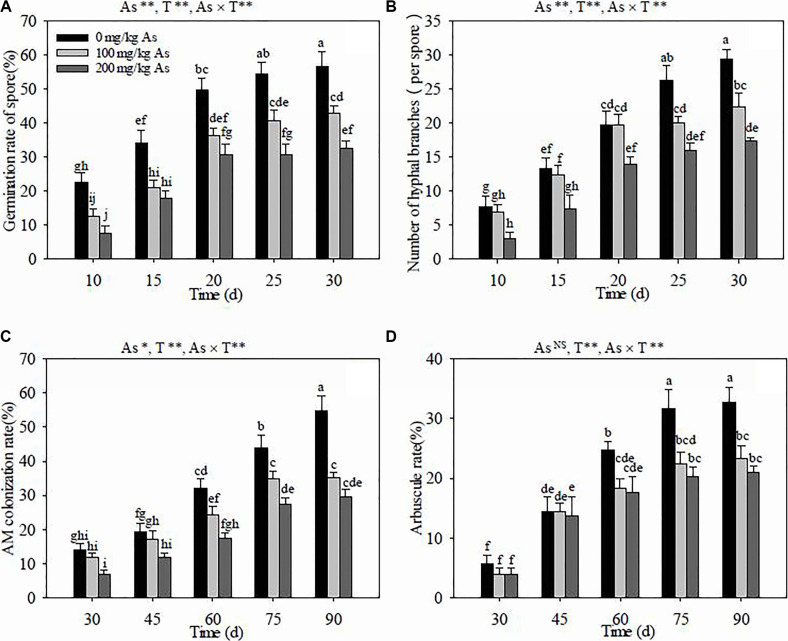
Effects of arsenic stress and culture (or colonization) time on the spore germination rate **(A)**, hyphal branch number **(B)**, arbuscular mycorrhiza (AM) colonization rate **(C)**, and arbuscule rate **(D)** of *Rhizoglomus intraradices*. *Different letters* indicate significant differences (*P* < 0.05) using Tukey’s test. Data are presented as the mean ± SD (*n* = 3). NS, not significant; As, arsenic addition levels; As×AMF, significant interactive effects of As level×*R. intraradices*; T, culture or colonization time. *P < 0.05; **P < 0.01.

Culture time had a noticeable effect on the germination of *R. intraradices* spores (*P* < 0.01), and the germination rate was gradually enhanced with the increase of culture time at the same As levels (Figure 1A). The As stress had a significantly negative effect on the germination rate (*P* < 0.01). The germination rate was obviously reduced by the increase of the As content in soils at the same culture time, except at 15 dpi where no difference was observed between the 100- and 200-mg kg^–1^ As levels. The results of the two-way ANOVA indicated that the germination rate of the AM spore was significantly influenced by the interaction between the As level in soils and the culture time (*P* < 0.01).

The number of hyphal branches in *R. intraradices* were obviously increased with culture time at the same As levels (*P* < 0.01) (Figure 1B), except those at 100 mg kg^–1^ As from 20 to 25 dpi. The As levels in soils remarkably decreased the branch number at the same culture time (*P* < 0.01), except for that at 20 dpi where the branch number was not significantly different between 0 and 100 mg kg^–1^ As. The results of the two-way ANOVA indicated that the number of hyphal branches exhibited highly significant differences with the interaction between the As concentration in soils and the culture time (*P* < 0.01).

### AM Colonization and Arbuscule Rate

The AM colonization rate in *R. pseudoacacia* roots was significantly enhanced along with colonization time at three different As levels (*P* < 0.01) (Figure 1C), except there was no obvious increase of the colonization rate after 75 dpi at 100 mg kg^–1^ As. The As levels in soils had a significantly negative influence (*P* < 0.05) on *R. intraradices* colonization in *R. pseudoacacia* roots. The AM colonization rate was the highest at 0 mg kg^–1^ As and gradually decreased with As concentrations at the same colonization time. The AM colonization rate was significantly affected by the interaction between the As level in soils and the inoculation time (*P* < 0.01).

The arbuscule rate was obviously increased along with colonization time, and the increase almost stagnated after 75 dpi at the three different As levels in soils (*P* < 0.01) (Figure 1D). The arbuscule rate was the highest at 0 mg kg^–1^ As compared to those at 100 and 200 mg kg^–1^ As after 60 dpi, but it had no apparent difference between 100 and 200 mg kg^–1^ As at 30, 60, and 90 dpi. The arbuscule rate indices were significantly affected by the interaction between the As concentration and the AM colonization time (*P* < 0.01), but not by the As levels in soils.

### Plant Biomass

**FIGURE 2:**
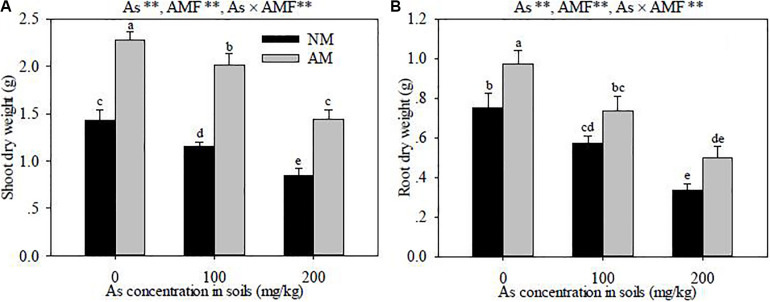
Effects of *Rhizoglomus intraradices* on the shoot **(A)** and root **(B)** dry weights in *Robinia pseudoacacia* seedlings under three different levels of As stress. *Different letters* indicate significant differences (*P* < 0.05) using Tukey’s test. Data are presented as the mean ± SD (*n* = 3). NS, not significant; As, arsenic addition levels; AMF, arbuscular mycorrhizal fungi inoculation; As×AMF, significant interactive effects of As level×*R. intraradices*. *P < 0.05; **P < 0.01.

Growing for 4 months, *R. pseudoacacia* seedlings exposed to As stress showed toxicity symptoms, which manifested in the lower shoot and root dry weights in As-contaminated soils (*P* < 0.01) (Figure 2). At the same As levels, the shoot and root dry weights in *R. intraradices*-inoculated seedlings were higher than those in the non-inoculated seedlings (*P* < 0.01). Significant interactive effects of As level × *R. intraradices* inoculation on the shoot and root dry weights were also found in this study (*P* < 0.01).

### Root Morphological Characteristics

**FIGURE 3:**
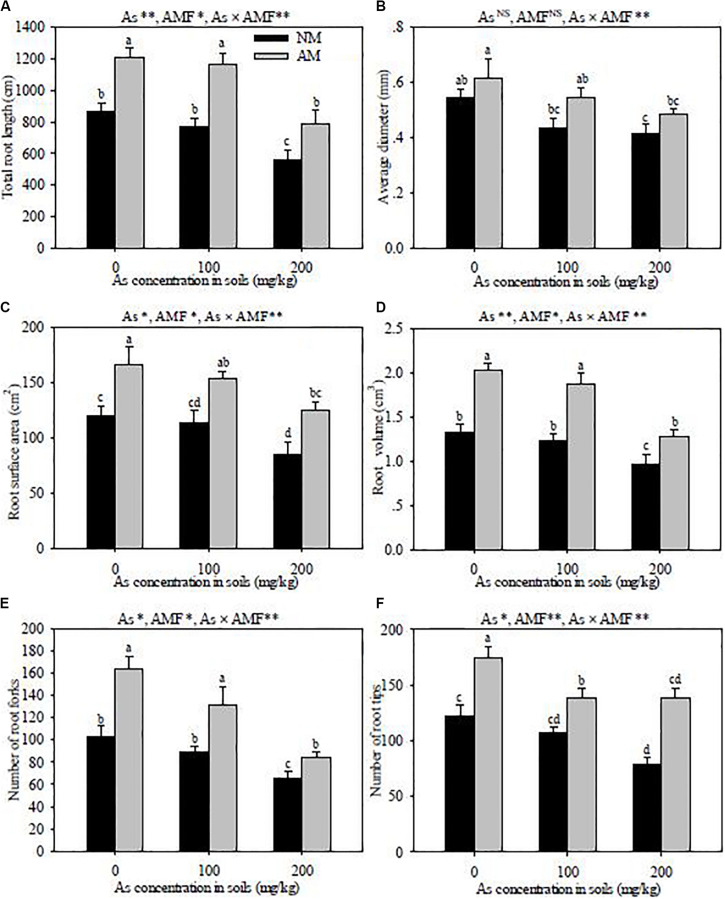
Effects of *Rhizoglomus intraradices* on total root length **(A)**, root average diameter **(B)**, root surface area **(C)**, root volume **(D)**, root fork number **(E)**, and root tip number **(F)** in *Robinia pseudoacacia* seedlings under three different levels of As stress. *Different letters* indicate significant differences (*P* < 0.05) using Tukey’s test. Data are presented as the mean ± SD (*n* = 3). NS, not significant; As, arsenic addition levels; AMF, arbuscular mycorrhizal fungi inoculation; As×AMF, significant interactive effects of As level×*R. intraradices*. *P < 0.05; **P < 0.01.

The increase of As concentration in soils obviously decreased the parameters of root morphological characteristics, including the total root length, root surface area, root volume, and the number of root forks and tips in both *R. intraradices-*inoculated and non-inoculated seedlings (Figure 3). *R. intraradices* inoculation alleviated the adverse effects of As stress, which improved the root diameter, total root length, root surface area, root volume, and the number of root forks and tips across all As treatments. Significant interactive effects of As level × *R. intraradices* inoculation on the total root length, average root diameter, root surface area, root volume, and the number of root forks and tips were detected in this experiment (*P* < 0.01).

**FIGURE 4:**
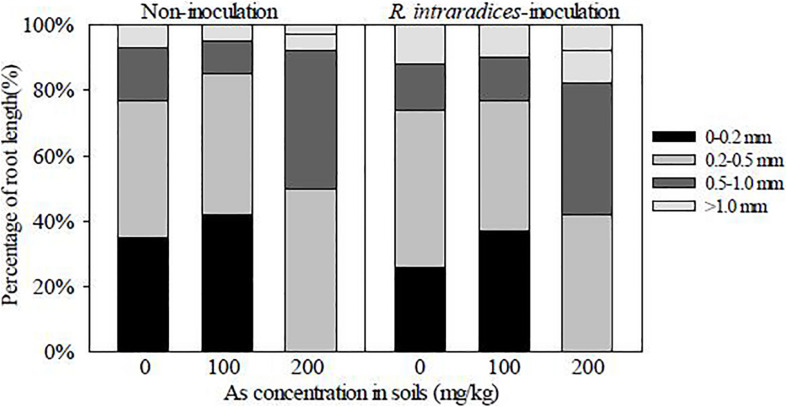
Percentages of root length in different diameter classes in *Rhizoglomus intraradices-*inoculated and non-inoculated *Robinia pseudoacacia* seedlings.

*R. intraradices* inoculation decreased the percentages of root length in the 0- to 0.2-mm diameter class in *R. pseudoacacia* seedlings, but increased those in the 0.5- to 1.0-mm and >1.0-mm diameter classes, compared with non-inoculation (Figure 4). With the increase of the As concentration in soils, the proportion in the 0- to 0.2-mm diameter class was increased, but those in the 0.5- to 1.0-mm and >1.0-mm diameter classes were suppressed. The percentages in the 0.2- to 0.5-mm diameter class were less affected by *R. intraradices* inoculation and As stress.

### T-GRSP and EE-GRSP in Soils

**FIGURE 5:**
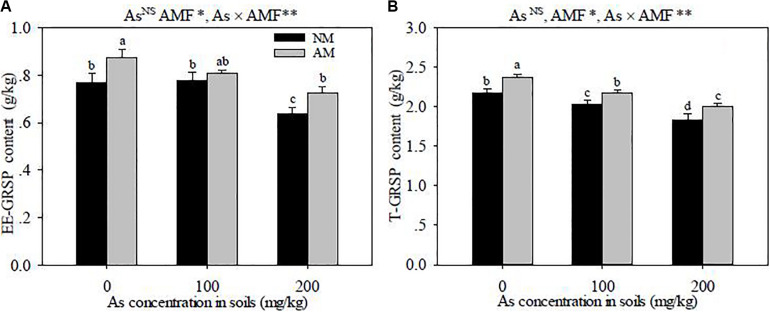
Effects of *Rhizoglomus intraradices* on the concentrations of EE-GRSP **(A)** and T-GRSP **(B)** in the rhizosphere soils of *Robinia pseudoacacia* seedlings under three different levels of As stress. *Different letters* indicate significant differences (*P* < 0.05) using Tukey’s test. Data are presented as the mean ± SD (*n* = 3). NS, not significant; As, arsenic addition levels; AMF, arbuscular mycorrhizal fungi inoculation; As×AMF, significant interactive effects of As level×*R. intraradices*; EE-GRSP, easily extractable glomalin-related soil protein; T-GRSP, total glomalin-related soil protein. *P < 0.05; **P < 0.01.

The EE-GRSP and T-GRSP contents were higher in the rhizosphere soils of *R. intraradices*-inoculated seedlings than those in the non-inoculated seedlings (*P* < 0.05) (Figure 5). The EE-GRSP and T-GRSP contents were decreased with the increase of As concentration in soils, except in the As levels between 0 and 100 mg kg^–1^ where there was no discernible difference in the contents of EE-GRSP. Significant interactive effects of As level × *R. intraradices* inoculation on the concentrations of EE-GRSP and T-GRSP were detected in this study (*P* < 0.01).

### Phytohormone Concentration and Ratio in Roots

**FIGURE 6:**
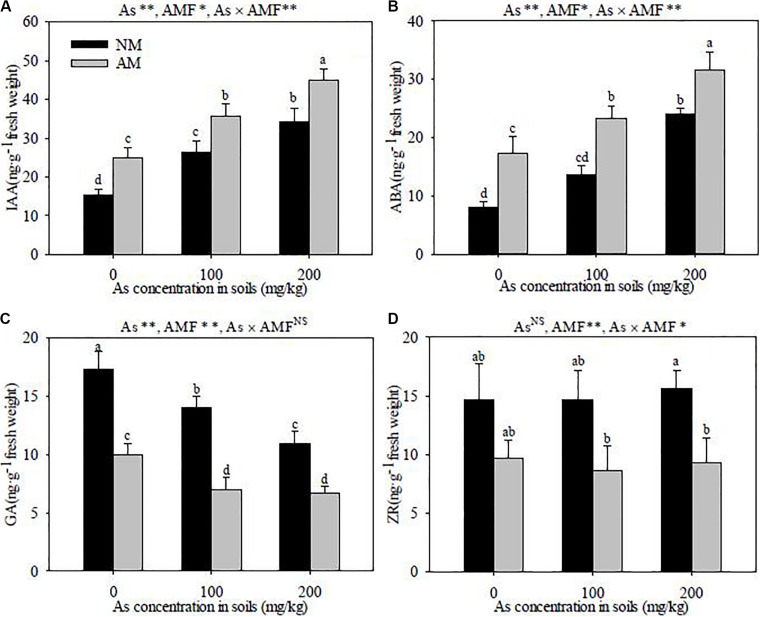
Effects of *R. intraradices* on the concentrations of IAA **(A)**, ABA **(B)**, GA **(C)**, and ZA **(D)** in *R. pseudoacacia* seedlings under three different levels of As stress Different letters indicate significant differences (*P* < 0.05) using Tukey’s test; Data is presented as mean ± SD (*n* = 3); NS, not significant; *P < 0.05; **P < 0.01; As, As addition levels; AMF, AMF inoculation; As×AMF, significant interactive effects of As level×*R. intraradices*; ABA, abscisic acid; GA, gibberellic acid; IAA, indole-3-acetic acid; ZR, zeatin riboside.

*R. intraradices* inoculation had notable effects on the concentrations of IAA, ABA, GA, and ZR in *R. pseudoacacia* roots. *R. intraradices* inoculation increased the concentrations of IAA and ABA (*P* < 0.05), but decreased GA and ZR under the same As levels (*P* < 0.01) (Figure 6). With the added level of As concentrations in the soils, the IAA and ABA contents were increased (*P* < 0.01) and GA was significantly decreased (*P* < 0.01), but it had no apparent effects on ZR in both *R. intraradices* inoculation and the non-inoculated *R. pseudoacacia*. Furthermore, significant interactive effects of As level × *R. intraradices* inoculation on the concentrations of IAA, ABA, and ZR were detected in this experiment (*P* < 0.05).

The phytohormone ratios of IAA/ABA, GA/ABA, ZR/ABA, and (IAA + GA + ZR)/IAA were significantly affected by *R. intraradices* inoculation, and these parameters in the *R. intraradices*-inoculated seedlings were lower than those of the non-inoculated ones under the same As levels (*P* < 0.05) (Table 1). The GA/ABA, ZR/ABA, and (IAA + GA + ZR)/IAA ratios were significantly decreased with the increase of As concentration in soils (*P* < 0.01), but the IAA/ABA ratio of the *R. intraradices*-inoculated seedlings was not affected by the As level. Significant interactive effects of the As level × *R. intraradices* inoculation on the phytohormone ratios of GA/ABA, ZR/ABA, and (IAA + GA + ZR)/IAA were also found in this study (*P* < 0.01).

**TABLE 1:** Effects of *Rhizoglomus intraradices* inoculation on the phytohormone ratios in *Robinia pseudoacacia* roots under different levels of As stress.

Inoculation	As(V) concentration (mg kg^–^^1^)	Hormone ratios			
		IAA/ABA	GA/ABA	ZR/ABA	(IAA + GA + ZR)/AB
Non-AMF	0	1.94 ± 0.13 a	2.18 ± 0.22 a	1.82 ± 0.16 a	5.94 ± 0.31 a
	100	1.95 ± 0.17 a	1.03 ± 0.08 b	1.07 ± 0.12 b	4.05 ± 0.51 b
	200	1.43 ± 0.09 b	0.46 ± 0.02 c	0.65 ± 0.08 c	2.54 ± 0.09 c
*R. intraradices*	0	1.47 ± 0.15 b	0.59 ± 0.12 cd	0.57 ± 0.15 cd	2.63 ± 0.25 c
	100	1.54 ± 0.16 b	0.30 ± 0.04 cd	0.37 ± 0.06 cd	2.21 ± 0.23 d
	200	1.43 ± 0.11 b	0.21 ± 0.04 d	0.29 ± 0.04 d	1.93 ± 0.09 d
Significance					
AMF		*	**	**	**
AS		NS	**	**	**
AMF × AS		NS	**	**	**

Different letters indicate significant differences (*P* < 0.05) using Tukey’s test. Data are presented as the mean ± SD (*n* = 3). As, arsenic addition levels; AMF, arbuscular mycorrhizal fungi; As×AMF, significant interactive effects of As level×*R. intraradices*; IAA, indole-3-acetic acid; ABA, abscisic acid; GA, gibberellic acid; ZR, zeatin riboside; NS, not significant. *P < 0.05; **P < 0.01.

## Discussion

In our study, *R. pseudoacacia* seedlings were cultivated in greenhouse pots and were subjected to three different levels of As stress with or without AMF *R. intraradices* inoculation. The germination rate of the AM spore, number of AM hyphal branches, plant growth, root morphological characteristics, phytohormone concentration and balance, and the GRSP content in rhizosphere soils were evaluated. The results revealed the possible mechanism that *R. intraradices* assisted *R. pseudoacacia* seedlings in enhancing tolerance to As toxicity. This study highlighted the possibility of vegetation recovery in As-contaminated areas by using AMF-inoculated woody legumes.

Previous research have found that AMF are colonized in the root cortex of *R. pseudoacacia* seedlings under various harsh environmental conditions, such as barren, salt, cold, drought, and Pb stress (Tian et al., 2003; Huang et al., 2019). In this study, we verified that *R. intraradices* could successfully establish symbioses with *R. pseudoacacia* seedlings under different levels of As concentrations in soils. The *R. intraradices* colonization rate was inhibited by the increase of As concentration in soils. This result was consistent with previous studies on other host species, such as *Cajanus cajan*, *Pisum sativum*, and *Glycine max* L. (Garg et al., 2015; Li et al., 2016; Spagnoletti and Lavado, 2015), but it was contrary to the conclusions of Leung et al. (2006). As stress in soils has negative effects on the AM colonization rate due to worsening of the pre-symbiotic conditions of mycorrhizal colonization with As toxicity (Spagnoletti et al., 2016). However, the AM colonization rate cannot be the only indicator in evaluating the responses of AMF on host plants (Li et al., 2016).

In the process of AMF–plants symbiotic association, the germinated AM spores undergo AM hyphal elongation and branching. The germination rate of the AM spore and the number of hyphal branches were the indicators of AM colonization development in plant roots (Délano-Frier and Tejeda-sartorius, 2008). The present work showed that the germination rate of the AM spore and the number of hyphal branches were reduced with increasing As concentration in soils. Ortega-Larrocea et al. (2007) have proven that the accumulation of toxic elements in soils could restrain the number of spores, germination, and AM hyphal colonization. Moreover, host plants often produce more root exudates, and they reduce the transfer of carbonaceous compounds to AMF under As stress conditions, which leads to the decrease of AM-colonized vitality in the root system (Spagnoletti et al., 2017).

Due to generating excessive reactive oxygen species (ROS) and disrupting the Calvin cycle of the photosynthetic system, As toxicity hindered plant growth, which could be alleviated by mycorrhizal symbioses (Chen et al., 2007). In this study, the shoot and root dry weights in *R. intraradices*-inoculated *R. pseudoacacia* seedlings were higher than those in the non-inoculated seedlings. *R. intraradices* inoculation was also found to increase the growth and yield in *G. max*, *Oryza sativa*, and *Triticum aestivum* in As-contaminated soils (Spagnoletti and Lavado, 2015; Li et al., 2016). As is an element of the phosphate analog and has the same transport pathway as phosphate across the plasma membrane of root cells (Chen et al., 2007). AM inoculation increased P nutrition and decreased As toxicity. The higher P/As content molar ratio in plants by AMF symbioses is one of the reasons for the improvement of plant growth (Li et al., 2016).

The parameters of root morphological characteristics, which included the root length, root surface area, root volume, and the number of root forks and tips, were also closely related to the adaptation, health status, and productivity of plants. The good morphological parameters indicated that the root system was more suitable for absorbing water and nutrients from soils (Huang et al., 2019). As a sensitive system of plants for perceiving the soil environment, the root tissue showed astonishing morphological changes to minimize the physiological metabolism and maximize the nutrient acquisition (Mishra et al., 2017). Root development is often hindered by HM toxicity, and the stagnation of root elongation was identified as the first evidence of any unfavorable influences (Munzuroglu and Geckil, 2002). In the current study, the total root length, root surface area, root volume, and the number of root forks and tips of *R. pseudoacacia* seedlings were decreased with the increase of As concentration in soils. Huang et al. (2019) also found that the Pb toxicity in soils decreased the root length, root diameter, and root surface area of *R. pseudoacacia* seedlings. Spagnoletti and Lavado (2015) verified that the morphological traits of *G. max* seedlings, like root length, were negatively influenced by the As concentration in soils. Srivastava et al. (2009) showed that the root density of *Brassica juncea* seedlings was slightly increased, but the length of the roots had no significant difference under As stress in soils. Such root modifications were a direct evidence of root damage caused by As toxicity in the soils and were most likely associated with a weakened plant metabolism (Chen et al., 2007).

The root length and root surface area, which are determined by the root length and the number of root forks and tips, are essential factors that determine the root volume. A higher number of root forks and tips indicates a better trend of root development (Liu and Wei, 2019). The symbiotic effects of AMF on the root system include the regulatory changes of root morphological characteristics in a textural, dimensional, quantificational, and impermanent manner (Sheng et al., 2009). This study showed that the root diameter, total root length, root surface area, root volume, and the number of root forks and tips of the *R. intraradices-*inoculated seedlings were higher than those of the non*-*inoculated seedlings under the same levels of As stress. Our results were similar to the conclusion by Huang et al. (2019) and Wu et al. (2011). The root lengths of *G. mosseae*-inoculated *Z. mays* were up to 3.4 times those of the non-inoculated controls in As-contaminated soils, and the enlarged root systems of the host plants also contributed primarily to nutrient acquisition with *G. mosseae* inoculation (Xia et al., 2007). Root tips are the closest part of plants in contact with soil substances, and the proliferation of root tips provides more opportunities for plants to acquire resources and nutrition from soils (Lu et al., 2013). In the present study, the number of root tips in *R. pseudoacacia* seedlings were decreased with the increase of the As level in soils; however, *R. intraradices*-inoculated seedlings had a higher number of root tips than the non-inoculated seedlings, which indicated that *R. intraradices* could alleviate the reduction in lateral root proliferation and facilitate the plant’s capacity for resource acquisition in As-contaminated soils. The probable explanation for our finding was that *R. intraradices* symbiosis was able to stimulate host plants to produce more fine roots in *R. pseudoacacia* seedlings under As stress conditions, which helped the root system of *R. pseudoacacia* seedlings to uptake more soil nutrient and water by increasing the active root length and the number of root forks and root tips.

In the present study, *R. intraradices*-inoculated seedlings had lower percentages of root length in the 0- to 0.2-mm diameter class, but higher percentages in the 0.5- to 1.0-mm and >1.0-mm diameter classes, compared with the non-inoculated seedlings. So *R. intraradices* inoculation increased the root diameter in *R. pseudoacacia* under different As treatment levels, which could probably be attributed to an enhanced parenchyma cell size and accrescent cortical tissues stimulated by AM symbiosis (Sheng et al., 2009). A similar result was also reported in *R. pseudoacacia* seedlings inoculated with *Rhizophagus irregularis* or *Glomus versiforme* in a pot experiment under normal growing conditions (Zhang et al., 2016). It indicated that the effects of AM inoculation on the percentage of root length in different diameter classes were generated not only under stress conditions but also under normal growth conditions. Zangaro et al. (2007) also found that the rootlet diameter of arboreal plants in South Brazil had a positive correlation with AM colonization. More prominent parameters of root diameter also indicated that the plant roots had higher penetration in soils (Liu and Wei, 2019). As toxicity damaged the morphology and configuration of *R. pseudoacacia* seedlings by forming shorter yet thicker main and lateral roots; however, *R. intraradices* inoculation improved the plant morphology by increasing the percentage of the thicker root length of *R. pseudoacacia* seedlings to better adapt to As stress. AMF played a crucial role in changing the root morphology, which should be extensively studied for improving phytoremediation efficiency in HM-contaminated areas.

Phytohormone is a class of trace secondary metabolites which plays irreplaceable roles in regulating plant traits (Bilal et al., 2019). Under HM stress condition, auxin (IAA) in roots often mediates morphological change of plants, especially stimulating the elongation and production of lateral roots (Ludwig-Müller, 2000). The root meristem constitutes the main site of HM action and also rapidly synthesizes a great deal of ABA. The inhibition of plant growth, which was induced by HM stress, is also in part a result of the increased ABA concentration (Liu and Wei, 2019). In this study, the contents of IAA and ABA were obviously increased. A similar result was also found in *B. juncea* seedlings under As toxicity stress (Srivastava et al., 2009). Various phytohormones mediated the downstream processing of stress perception; as a result, As stress was perceived by plants (Srivastava et al., 2009).

Plants and AMF can form a beneficial symbiotic relationship by establishing an intricate network of signaling and biochemical pathways (Délano-Frier and Tejeda-sartorius, 2008) in which AMF-regulated phytohormones are suitable candidates for continuous signal exchanges between plant cells and the AM hyphae (Liu and Wei, 2019). These phytohormones regulated the plant metabolic and structural genes, whose products, in return, affected the symbiosis between the AMF and plants; ultimately, plant growth is enhanced by AMF-regulated phytohormones (Ludwig-Müller, 2000). In this study, *R. intraradices* inoculation increased the IAA and ABA concentrations and decreased the GA and ZR contents in *R. pseudoacacia* seedlings. Similar results were reported by Bilal et al. (2019), in which endophytic *Penicillium funiculosum* inoculation decreased the ABA content in *G. max* seedlings due to downregulating the HM ATPase genes under combined HM stress. Zahoor et al. (2017) reported that phytohormones induced by the endophyte *Mucor* sp. could help *Brassica campestris* alleviate HM toxicity. Müller and Harrison (2019) also verified that phytohormones participated in the AM-associated effects on plant morphology, growth, and stress resistance. In this study, *R. intraradices* inoculation enhanced the ABA and IAA contents under As stress, which resulted in promoting stomatal closure, decreasing transpiration in host roots, coordinating the balance of growth-related hormones to plant growth, and, ultimately, increasing the tolerance of *R. pseudoacacia* seedlings to As toxicity.

Physiological processes (for example, phytohormone metabolism) were forced to change by As stress in soils, but AMF symbiosis helped host plants to better adapt to environmental stress by regulating the content and ratio of phytohormone. It was suggested that hormones, released by mycorrhizal fungi, may contribute to the enhancement of plant growth (Frankenberger and Arshad, 1995). Phytohormones responded synergistically to HM stress conditions by regulating their synthetic ability, changing the hormone content, and adjusting the balance and ratio of hormones, in which the ratios of the stress-related phytohormones often have more profound implications than does the role of a single phytohormone in plants (Liu and Wei, 2019). Barker and Tagu (2000) verified that changes in the phytohormone ratios affected the different stages of plant development, which were closely related to mycorrhizal symbiosis. Berta et al. (1993) found that AM colonization could alter the ratio of hormones in host plants, as a result influencing the meristem activity in the root apex. In the present study, the GA/ABA, ZR/ABA, and (IAA + GA + ZR)/IAA ratios in *R. pseudoacacia* seedlings were also significantly decreased with the increased level of As concentration in soils; however, the phytohormone ratios of IAA/ABA, GA/ABA, ZR/ABA, and (IAA + GA + ZR)/IAA in *R. intraradices*-inoculated seedlings were lower than those in the non-inoculated seedlings under the same As levels. The IAA/ABA ratios of *Acrocalymma vagum*-inoculated *Ormosia hosiei* seedlings were higher than those of the non-inoculated seedlings, but the ratios of GA/ABA, ZR/ABA, and ZR/IAA of *A. vagum*-inoculated seedlings were lower than those of the non-inoculated seedlings under drought stress conditions (Liu and Wei, 2019). Our results showed that *R. intraradices* symbiosis with *R. pseudoacacia* seedlings coordinated the balance of phytohormones through adjusting the hormone ratios under As stress, as a result slowing down the plant growth and development, reducing plant respiration, maintaining normal water balance, and improving osmotic pressure in plant cells. Thereby, the resistance of *R. pseudoacacia* seedlings to As toxicity was enhanced. However, the mechanism of AMF regulating the production and balance of phytohormones under As stress needs to be further elaborated from the level of molecular biology.

The AM extraradical hyphae secrete a kind of insoluble hydrophobic protein, i.e., glomalin, which increases soil fertility and keeps the porous and moist soil structure for the optimal growth of plants (Rillig, 2004). Glomalin is quantified as glomalin-related soil protein (GRSP), and the GRSP content in soils is often used as a criterion to evaluate the soil capacity for nutrient storage and water-holding (Wright and Upadhyaya, 1996; Rillig, 2004). In this study, the contents of EE-GRSP and T-GRSP were decreased with the increase of As concentration in soils; this result was contrary to the previous research by González-Chávez et al. (2009), who considered that the accumulation of GRSP in the soils was not affected by the increase of Pb or Zn concentration. Glomalin binds HM ions (like Cu, Cd, Pb, and Zn) and immobilizes them by adsorbing to the cell walls of the AM hyphae (Spagnoletti et al., 2017). Bedini et al. (2009) first found a cause–effect relationship between AM symbiosis and GRSP content in rhizosphere soils. Some evidence have shown that there is a higher correlation between GRSP and the length of AM extraradical hyphae: a high glomalin content corresponds to further expansion of AM extraradical hyphae (Lovelock et al., 2004; Zhang et al., 2016). In this work, *R. intraradices* inoculation obviously increased the contents of EE-GRSP and T-GRSP in soils under the same As treatments. It was reasonable to speculate that the increased GRSP content contributed to the incremental length of AM extraradical hyphae and the rapid turnover time of the AM hyphae compared with the no-inoculation treatment. Hence, the As resistance of *R. pseudoacacia* seedlings was enhanced by the AMF-secreted glomalin.

## Conclusion

As stress toxicity suppressed the AM spore germination and colonization, plant growth, and the content of soil glomalin and changed the morphological characteristics of the roots and the balance of endogenous hormone levels in plants. However, the shoot and root dry weights, the percentages of root length in the 0.5- to 1.0-mm and >1.0-mm diameter classes, the IAA and ABA concentrations in *R. pseudoacacia* seedlings, and the contents of EE-GRSP and T-GRSP in rhizosphere soils were increased. The total root length, root surface area, root volume, and the number of root forks and tips were significantly improved by *R. intraradices* inoculation. *R. intraradices* inoculation also decreased the percentages of root length in the 0- to 0.2-mm diameter class, the GA and ZR concentrations, and the phytohormone ratios [IAA/ABA, GA/ABA, ZR/ABA, (IAA + GA + ZR)/IAA] across the different As treatments compared with non-inoculation. Our results demonstrated that *R. intraradices* inoculation alleviated As toxicity in *R. pseudoacacia* seedlings by improving the plant growth, altering the root morphology, regulating the concentrations and ratios of phytohormones, and increasing the concentration of soil glomalin. Mycorrhizal symbioses played a crucial role in revegetation and ecological remediation in As-contaminated areas.

## Data Availability Statement

All datasets presented in this study are included in the article/supplementary material.

## Author Contributions

QZ and KL conducted the experiments and collected the data. QZ, YC, and JY drafted and revised the manuscript. KL, YC, and JY participated in data set assessment, script preparation, and manuscript revision. MG was the principal investigator who designed the study and finalized the script. All authors contributed to the article and approved the submitted version.

## Conflict of Interest

The authors declare that the research was conducted in the absence of any commercial or financial relationships that could be construed as a potential conflict of interest.
